# Investigating Reinforcing and Cracking Resistance Behaviors of Waste Sweet Potato Vine Straw Fiber (WSVF) in Gel-like Base Asphalt

**DOI:** 10.3390/gels12030239

**Published:** 2026-03-13

**Authors:** Chenze Fang, Zhenxia Li, Yuanzhao Chen, Xu Guo, Hui Li, Naisheng Guo, Zongyuan Wu, Jingyu Yang, Tengteng Guo

**Affiliations:** 1 School of Civil Engineering and Transportation, North China University of Water Resources and Electric Power, Zhengzhou 450045, China; 2 Technology Innovation Center of Henan Transport Industry of Utilization of Solid Waste Resources in Trafffc Engineering, North China University of Water Resources and Electric Power, Zhengzhou 450045, China; 3 Henan Province Engineering Technology Research Center of Environment Friendly and High-Performance Pavement Materials, Zhengzhou 450045, China; 4 Department of Transportation, Southeast University, Nanjing 210096, China; huili94@seu.edu.cn; 5 Department of Transportation Engineering, Dalian Maritime University, Dalian 116026, China

**Keywords:** road engineering, waste sweet potato vine straw fiber, reinforcing and cracking resistance, cracking volume, damage variable, activation energy

## Abstract

Waste sweet potato vine fiber (WSVF) effectively extends asphalt service life by enhancing cracking resistance in gel-like base asphalt matrices, yet its crack-resistant mechanism lacks mechanical characterization. This study proposes an analytical method for evaluating WSVF-modified asphalt’s crack-resistant behavior based on the principle of mechanical energy balance. First, alkali-treated WSVF with a mass fraction of 1% was added into 70# gel-like base asphalt to prepare WSVF-modified asphalt. Lignin fiber (LF)-modified asphalt and 70# gel-like base asphalt were selected as control groups, and three types of time sweep and scanning electron microscopy tests were conducted. Then, the three-dimensional cracking volume model and damage kinetics model were established for analyzing the cracking response behavior, defining the asphalt damage variable and determining the cracking damage activation energy (*E*_acd_). Finally, the *E*_acd_ was used to quantify the difficulty of the cracking damage process for the WSVF-modified asphalt. The reinforcement and cracking resistance mechanisms of WSVF in asphalt were analyzed by the *E*_acd_ and asphalt microstructure. The results show that the cracking volume response of WSVF-modified asphalt under cyclic loading presents three-stage nonlinear behaviors. The established fatigue damage kinetics model can accurately describe the fatigue damage evolution process of alkali-treated WSVF-modified asphalt. The *E*_acd_ values of WSVF-modified asphalt, LF-modified asphalt, and 70# gel-like base asphalt are 10.60 kJ·mol^−1^, 21.83 kJ·mol^−1^, and 29.74 kJ·mol^−1^, respectively. After alkali treatment, the WSVF surface exhibits grooves, demonstrating superior adsorption and storage capacity for asphalt. The WSVF can cross link through the bonding effect of asphalt and form a three-dimensional network framework structure, which can significantly increase the *E*_acd_ and provide strengthening and toughening effects on gel-like base asphalt. In summary, *E*_acd_ values are used as a mechanical indicator to quantitatively evaluate the fatigue cracking resistance of WSVF-modified asphalt.

## 1. Introduction

The annual global production of crop straw exceeds 5 billion tons, but the utilization efficiency is low. At present, it is mainly used for burning and fertilizer, and its application in road traffic materials is still limited [1,2,3]. Taking the large amount of surplus waste sweet potato vine straw fiber (WSVF) as an example, it is mostly burned, or simply returned to the field. Developing WSVF into road reinforcement fiber can promote diversified and high-value use of straw resources, and also help reduce environmental pollution, showing significant resource and environmental benefits.

According to classical colloid theory, conventional base asphalt typically exhibits characteristic gel-like structural features, which ensure its excellent high-temperature stability and low-temperature crack resistance. As an effective asphalt performance modifier, the effect of fiber mainly depends on the fiber type, dosage, and intrinsic properties. These parameters govern the extent to which fibers interact with and modify the asphalt matrix, thereby exerting a decisive influence on the overall performance of asphalt composites, including resistance to permanent deformation at elevated temperatures, fracture behavior under low-temperature conditions, and fatigue durability, as well as moisture-induced damage resistance [4,5,6]. Yang et al. [7] established an asphalt–basalt fiber interfacial model and found that the adhesion performance at the basalt fiber–asphalt interface was relatively stable. Li et al. [8] found that incorporating polyester fiber into asphalt mixtures significantly improved both toughness and water stability. Xie et al. [9] found that glass fiber could significantly improve asphalt performance, primarily through adsorption effects and reinforcement mechanisms. Existing studies have clearly demonstrated that fibers, when used as asphalt modifiers, can enhance the pavement performance of asphalt mixtures. Existing studies have confirmed that fiber, as an asphalt modifier, can improve the road performance of asphalt mixtures. Based on a deeper understanding of the fiber modification mechanism, finding fiber materials with wide sources and environmental friendliness has become an important research direction.

Plant fiber, as a natural fiber with wide sources, low cost, and biodegradability, is considered a promising modification material for asphalt pavement because of its good overall properties [10,11,12]. Khan et al. [13] incorporated coconut fiber into hot-mix asphalt, with results indicating it effectively enhances the mixture’s road performance. Li et al. [14] studied corn straw fiber asphalt mixture and found that the fibers physically blend and reinforce the asphalt, improving its performance at the microscopic level, and, thus, significantly enhancing the overall road performance of the mixture. Lei et al. [15] verified that the incorporation of cotton straw fiber can significantly enhance the high-temperature stability and low-temperature cracking resistance of SMA-13 and AC-13 mixtures, while also improving their water stability. However, the abundance of hydroxyl groups on the surface of plant fibers imparts strong hydrophilicity, resulting in poor interfacial compatibility with asphalt. To address this issue, surface modification of the fibers is typically required. By increasing surface roughness and specific surface area, the adsorption capacity of fibers for asphalt can be effectively enhanced, thereby improving their dispersion uniformity within asphalt mixtures [16]. Zhu et al. [17] found that alkaline solution pretreatment significantly enhanced the surface wettability, electrical conductivity, and diffusion coefficient of straw, and these improvements in interfacial properties, in turn, markedly promoted the penetration of resin into the interior of the straw. Li et al. [18] showed that treating reed with lignin-degrading microorganisms at pH = 8.0 resulted in the degradation of approximately 70% of lignin and 43% of hemicellulose, thereby increasing the relative content of cellulose. Li et al. [19] systematically compared the micro-morphology, chemical composition, and thermal stability of sweet potato vine straw fiber prepared using physical treatment, NaOH solution treatment, and HCl solution treatment, and ultimately identified the NaOH solution method as the optimal preparation process. In summary, WSVF can serve as a safe and effective asphalt modifier for improving the service performance of asphalt materials.

The selection of WSVF as a modifier for gel-like matrix asphalt is primarily based on its favorable physicochemical properties and the potential for high-value utilization of agricultural waste. WSVF primarily consists of cellulose, hemicellulose, and lignin, with cellulose content typically ranging between 35% and 45%, conferring high tensile strength and thermal stability to the fibers. Furthermore, appropriate alkali treatment can significantly increase the fiber’s aspect ratio and enhance surface grooves and roughness. These characteristics collectively improve the fiber’s asphalt absorption capacity and interfacial adhesion with the asphalt matrix [19]. WSVF serves not only as a renewable, low-cost reinforcing agent, but also as a structural–functional component capable of modifying asphalt’s viscoelastic response and toughening behavior. Although the above studies have provided a theoretical basis for the road engineering reutilization of sweet potato vine straw, existing research has mainly focused on treatment processes of the straw and the rheological properties of the modified asphalt. In particular, systematic and in-depth mechanical characterization of the cracking damage behavior of WSVF-modified asphalt under cyclic loading remains lacking. In addition, damage variable definition methods based on traditional phenomenological indices (e.g., modulus and strength degradation) lack rigorous mechanical derivation [20,21,22]. They are unable to capture the significant differences in mechanical response between damaged and undamaged asphalt, and make it difficult to accurately define the damage variables of WSVF-modified asphalt, resulting in a lack of a scientifically rigorous mechanical explanation for the toughening mechanism of WSVF in asphalt. Meanwhile, existing studies [23,24,25] have shown that the energy–mechanics approach can accurately describe the damage behavior of asphalt from a fundamental mechanical perspective by establishing energy–mechanics balance equations for damaged and undamaged asphalt. Given these concerns, this study aims to derive a cracking volume indicator with clear mechanical significance using the energy–mechanics approach and to investigate the toughening effect of the alkali-treated WSVF-modified asphalt based on cracking volume. First, a cracking volume model for the alkali-treated WSVF-modified asphalt is established. Then, a damage kinetics model for alkali-treated WSVF-modified asphalt is developed to determine the representative rates of the cracking damage process and cracking damage activation energy. Finally, the toughening mechanism of WSVF on 70# gel-like base asphalt is characterized by comparing the crack damage activation energy of the alkali-treated WSVF-modified asphalt and base asphalt. The objective of this study is to employ the energy–mechanics approach to derive a cracking volume indicator with well-defined mechanical significance, and to investigate the toughening and cracking resistance mechanism of alkali-treated waste sweet potato vine straw fiber (WSVF) in gel-like base asphalt based on the cracking volume. The main novelty of this study lies in (1) establishing a cracking volume model derived from energy–mechanical balance equations to directly quantify asphalt damage; (2) developing a fatigue damage kinetics model that incorporates the damage activation energy (*E*_acd_) parameter based on kinetics theory, enabling quantitative evaluation of the cracking damage process difficulty; and (3) revealing the microstructural toughening mechanism of WSVF through the correlation between fiber surface characteristics and the enhanced activation energy. This proposed evaluation method provides a scientific basis for extending asphalt pavement service life through the valorization of agricultural waste resources.

## 2. Results and Discussion

### 2.1. Fatigue Cracking Behavior Characterization of Alkali-Treated WSVF-Modified Asphalt Based on the Energy–Mechanical Approach

#### 2.1.1. Development of an Asphalt Cracking Volume Model Based on the Energy–Mechanical Approach

**Figure 1:**
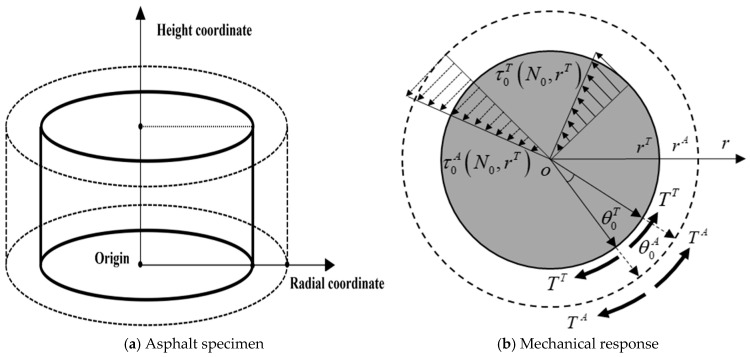
Schematic diagrams of asphalt specimen and its mechanical response during cyclic loading process.

Figure 1 illustrates the asphalt specimen and the corresponding stress state under cyclic shear loading. The entire asphalt specimen (the cylinder indicated by dashed lines) consists of two parts: the cracked and damaged asphalt region (the annular zone denoted by dashed and solid lines) and the uncracked, undamaged asphalt region (the solid-line cylinder). During the loading process, cracking damage initiates at the outer peripheral edge of the specimen and gradually propagates and extends toward the specimen center. Although the actual boundary between cracked and intact asphalt is irregular, it is idealized as a concentric circular interface based on the equivalent area principle to enable analytical derivation. The limitations include: (1) the complex energy–mechanical derivation and the need for additional non-damage tests limit its direct engineering applicability; (2) the idealized annular cracking pattern may lead to deviations between the calculated crack volume and the true physical crack volume; and (3) although the true modulus *G^T^* is approximated by NDTS testing at *τ_cri_*, it remains susceptible to nonlinearity or hardening effects. The assumption of constant fatigue modulus throughout the cycle neglects the evolution of the matrix modulus, potentially introducing systematic errors in long-term crack volume estimation.

The entire asphalt specimen is defined as the apparent asphalt, while the uncracked and undamaged asphalt is defined as the true asphalt, and their mechanical responses are illustrated in Figure 1b. During cyclic shear loading, the shear stress and shear strain of the apparent asphalt are expressed by Equations (1) and (2), respectively.

(1) $$\tau^{A}\left(t,r\right)=\tau_{0}^{A}\left(t_{0},r\right)\sin\left(\omega t\right)$$

(2) $$\gamma^{A}\left(t,r\right)=\gamma_{0}^{A}\left(t_{0},r\right)\sin\left(\omega t-\delta \right)$$

where *τ^A^*(*t*,*r*) and *γ^A^*(*t*,*r*) represent the stress and strain induced in the apparent asphalt, respectively; *t* denotes loading time; *r* denotes specimen radius length; *t*_0_ denotes at initial time; $\tau_{0}^{A}\left(t_{0},r\right)$ and $\gamma_{0}^{A}\left(t_{0},r\right)$ denote shear stress amplitude and shear strain amplitude, respectively; *ω* denotes angular velocity; and *δ* denotes phase angle.

For the true asphalt during cyclic shearing process, its stress and strain are expressed by Equations (3) and (4).

(3) $$\tau^{T}\left(t,r\right)=\tau_{0}^{T}\left(t_{0},r\right)\sin\left(\omega t\right)$$

(4) $$\gamma^{T}\left(t,r\right)=\gamma_{0}^{T}\left(t_{0},r\right)\sin\left(\omega t-\delta \right)$$

where *τ^T^*(*t*,*r*) and $\tau_{0}^{T}\left(t_{0},r\right)$ denote the shear stress and its amplitude of true asphalt, respectively; and *γ^T^*(*t*,*r*) and $\gamma_{0}^{T}\left(t_{0},r\right)$ denote the shear strain and its amplitude of true asphalt, respectively.

Although the cracking damage asphalt can lead to significant differences in the mechanical response between true asphalt and apparent asphalt, relevant studies [26] indicate that the two exhibit energy–mechanical equilibrium equations in terms of recoverable strain energy, dissipated strain energy, and torque, as shown in Equations (5)–(7).

(5) $$RSE^{T}=RSE^{A}$$

(6) $$DSE^{T}=DSE^{A}$$

(7) $$T^{T}=T^{A}$$

where *RSE^T^* and *RSE^A^* represent recoverable strain energy of true asphalt and apparent asphalt, respectively; *DSE^T^* and *DSE^A^* represent dissipated strain energy of true asphalt and apparent asphalt, respectively; and *T^T^* and *T^A^* represent torque of true asphalt and apparent asphalt, respectively.

By substituting the apparent and true shear strain models and shear stress models into torque balance Equation (5) and energy balance Equations (6) and (7), the fatigue crack length of the asphalt specimen can be determined. The expression is as follows [27,28].

(8) $$c=\left[\begin{array}{c} 1-{\left(\frac{|G^{*A}|}{|G^{*T}|}\right)}^{1/4} \end{array}\right]r^{A}$$

In the equation, *c* represents the fatigue crack length; |G*^A^| denotes the apparent shear modulus of the material; and |G*^T^| indicates the true shear modulus of the material.

(9) $$S=\pi {\left(r^{A}\right)}^{2}-\pi {\left(r^{A}-c\right)}^{2}$$

In the equation, *S* represents the crack area; *r^A^* denotes the apparent asphalt radius, numerically equivalent to the radius of the entire asphalt specimen; and *c* represents the fatigue crack length.

By combining the above three energy–mechanical equilibrium equations, a cracking volume model for asphalt can be derived, as shown in Equation (10). Equation (10) indicates that modulus decay can quantitatively reflect the cracking volume of asphalt. Since the cracking volume provides an intuitive quantification of asphalt damage, this study will analyze the damage evolution of asphalt during repeated loading based on this cracking volume model.

(10) $$CV=\pi {\left(r^{A}\right)}^{2}\left\{1-\left[1-{\left(1-{G_{r}}^{0.25}\right)}^{2}\right]\right\}h$$

where *CV* is cracking volume of asphalt; *r^A^* is apparent asphalt radius, numerically equivalent to the radius of entire asphalt specimen; *G_r_* is the modulus ratio, numerically equal to the shear modulus corresponding to apparent asphalt divided by that corresponding to true asphalt; and h is the cracking height of asphalt.

**Figure 2:**
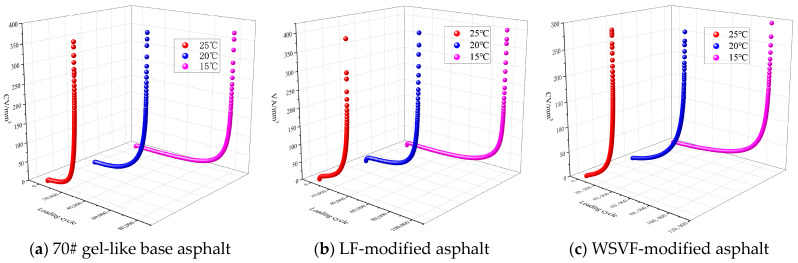
Cracking volume evolution curves of WSVF-modified asphalt.

Based on the cracking volume model derived from Equation (8), the cracking volume results of the WSVF-modified asphalt, LF-modified asphalt, and 70# gel-like base asphalt were obtained, as shown in Figure 2. As illustrated in Figure 2, under intermediate temperature conditions of 25 °C, 20 °C, and 15 °C, the cracking volume evolution curves of the WSVF-modified asphalt, the LF-modified asphalt, and 70# gel-like base asphalt under cyclic loading all exhibit similar nonlinear accumulation trends. Comparing Figure 2a–c clearly shows that, under identical temperature and stress conditions, both WSVF-modified asphalt and LF-modified asphalt exhibit slower overall trends in crack volume growth compared to 70# gel-like matrix asphalt. The retarding effect of WSVF-modified asphalt is particularly pronounced. This phenomenon is particularly evident during the mid-to-late loading stages (corresponding to the second and third phases), where the cracking volume curve of WSVF-modified asphalt consistently remains below those of LF-modified asphalt and base asphalt. This indicates that the incorporation of WSVF more effectively delays crack propagation.

#### 2.1.2. Cracking Volume Analysis of Alkali-Treated WSVF-Modified Asphalt

**Figure 3:**
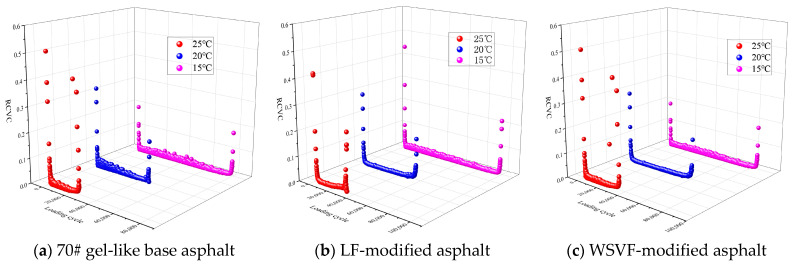
Crack propagation rate evolution curves of WSVF-modified asphalt.

To quantitatively analyze the evolution of cracking volume in the alkali-treated WSVF-modified asphalt, the rate of cracking volume change (*RCVC*) was defined, as expressed in Equation (11). Based on Equations (10) and (11), the relative variation curves of cracking volume and the *RCVC* for the WSVF-modified asphalt, LF-modified asphalt, and s70# gel-like base asphalt under constant-amplitude loading were obtained, as shown in Figure 3.

(11) $$RCAC=\frac{\left|CA_{b}-CA_{a}\right|}{CA_{a}(b-a)}$$

where *RCVC* denotes the rate of crack volume change in asphalt; *VA_a_* represents the cracking volume of asphalt at the ath cycle; and *VA_b_* represents the cracking volume of asphalt at the *b*-th cycle.

Analysis of Figure 3 shows that the cracking volume responses of the alkali-treated WSVF-modified asphalt, LF-modified asphalt, and 70# gel-like base asphalt throughout the cyclic shear process can be sequentially divided into three nonlinear stages: a rapid initial accumulation stage, a long-term constant-rate fluctuation stage, and a short-term accelerated growth stage. In the rapid initial accumulation stage, the cracking volume quickly increases from zero to a certain level, while the cracking volume growth rate continuously decreases. Upon entering the long-term constant-rate fluctuation stage, the cracking volume maintains a stable accumulation trend, and the cracking volume growth rate remains nearly constant. During this stage, the asphalt exhibits stable resistance to fatigue cracking. In the final short-term accelerated growth stage, the cracking volume continues to accumulate, and the cracking volume growth rate increases continuously. Ultimately, the alkali-treated WSVF-modified asphalt, LF-modified asphalt, and 70# gel-like base asphalt lose their resistance to fatigue cracking and undergo fatigue failure. From the above analysis, it can be concluded that the cracking volume growth rate can effectively reflect the fatigue cracking resistance of asphalt, and a negative correlation exists between them. Therefore, in this study, the intersection point of the tangents to the cracking volume growth rate curve at the long-term constant-rate fluctuation stage and the short-term accelerated growth stage is used to define the fatigue life (*N_f_*) of asphalt. Compared to both WSVF-modified asphalt and LF-modified asphalt, the *RCVC* curve of 70# gel-like base asphalt enters the steeply rising third stage the earliest. This phenomenon indicates that, under identical cyclic loading, the base asphalt loses its fatigue crack resistance most rapidly, thereby reaching fatigue failure sooner. In contrast, the modified asphalts delay the transition to the failure stage, exhibiting more enduring and stable crack resistance. Notably, the WSVF-modified asphalt demonstrates a more pronounced delay than the LF-modified asphalt, confirming that the incorporation of waste sweet potato vine straw fiber provides superior enhancement of fatigue crack resistance in base asphalt.

The fatigue life results of the alkali-treated WSVF-modified asphalt and 70# gel-like base asphalt are listed in Table 1. As shown in Table 1, at temperatures of 15 °C, 20 °C, and 25 °C, the corresponding load amplitudes were 250 kPa, 100 kPa, and 80 kPa, respectively, and the fatigue lives of the 70# gel-like base asphalt were 79,410; 45,040; and 25,800 cycles. Under the same conditions, the fatigue lives of the LF-modified asphalt were 97,880; 55,466; and 32,022 cycles, respectively, while those of the alkali-treated WSVF-modified asphalt were 105,880; 60,466; and 34,422 cycles, respectively. These results indicate that the incorporation of WSVF and LF into the 70# gel-like base asphalt can effectively retard crack propagation and extend the service life of asphalt. The average increases in fatigue life provided by WSVF and LF are approximately 23% and 34%, respectively, demonstrating that both fibers have significant toughening effects on asphalt.

### 2.2. Microstructural Morphology Analysis of Alkali-Treated WSVF-Modified Asphalt

**Figure 4:**
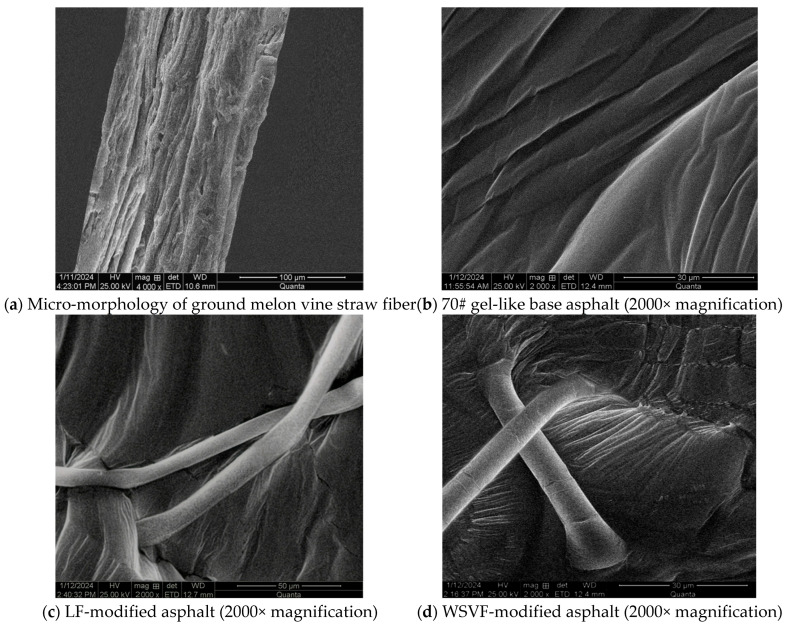
Micro-morphologies of different types of fibers in fiber-modified asphalt.

The micro-morphologies of the alkali-treated WSVF and the alkali-treated WSVF-modified asphalt obtained from SEM are shown in Figure 4. As can be observed from Figure 4, a large amount of asphalt adheres to and coats the surfaces of both LF and WSVF. The surface of lignin fiber without chemical solution treatment is relatively smooth, and its surface roughness is significantly lower than that of WSVF. This difference in surface morphology directly affects their interfacial bonding performance with asphalt. Owing to its higher surface roughness, WSVF can bond more effectively with asphalt, thereby exhibiting superior asphalt adsorption and storage capacity. After NaOH treatment, most of the fragmentary impurities on the surface of WSVF are removed, and a small number of grooves are observed on the fiber surface. These grooves promote the separation of fiber bundles, enhance the oil absorption capacity of the fibers, and effectively remove pectin and other impurities contained within the fibers, indicating that the NaOH treatment reduces the fiber diameter and increases the aspect ratio. From the local detailed observations in Figure 4, it can be further seen that the alkali-treated WSVF exhibits grooved surface structures, which provide excellent asphalt adsorption and storage capability and lead to tight interfacial bonding between the fiber and asphalt. In the modified asphalt system, the fibers overlap and interconnect through the adhesive action of asphalt to form a three-dimensional network skeleton structure, which can effectively transfer stress and inhibit crack propagation. As a result, WSVF plays a significant reinforcing and toughening role in asphalt.

### 2.3. Development of a Fatigue Damage Kinetics Model for Alkali-Treated WSVF-Modified Asphalt

Compared with indirect indicators of asphalt damage, such as modulus decay and energy dissipation, the cracking volume has a clear physical meaning and can directly quantify the damage level of asphalt. Therefore, in this study, the ratio of the cracking volume to the total volume of the asphalt specimen is defined as the asphalt damage variable, as expressed in Equation (12).

(12) $$D=\frac{CA_{N}}{SA}$$

where *D* is damage variable; *VA_N_* is specimen cracking volume of the *N*-th cycle; and *SA* is specimen volume, numerically equal to *π*(*r^A^*)^2^h.

**Figure 5:**
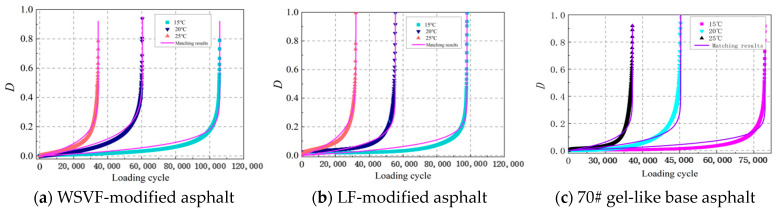
Fatigue damage curves of asphalt under constant amplitude.

The asphalt damage curves determined using Equation (13) are shown in Figure 5. As illustrated in Figure 5, under cyclic shear loading, the damage evolution of the alkali-treated WSVF-modified asphalt, the LF-modified asphalt, and 70# gel-like base asphalt exhibits an overall trend of increasing damage rate. Previous studies [29] have indicated that the damage evolution rate of viscoelastic materials can be characterized by the evolution rate of dissipated energy, as follows:

(13) $$\frac{dD}{dN}={\left(\frac{\partial W_{N}}{\partial N}\right)}^{\beta }$$

where W denotes the dissipated energy, in J/m^3^, and *β* is a material parameter.

The cracking damage process of asphalt under cyclic loading is a complex physical process characterized by a varying evolution rate. To quantify the rate at which this process occurs, the logarithm of Equation (13) is taken, yielding:

(14) $$D=1-{\left(1-\frac{N}{N_{f}}\right)}^{\frac{1}{1+2\beta }}$$

Analysis of Equation (14) indicates that a linear relationship exists between the evolution of dissipated energy and damage evolution in logarithmic coordinates, and the slope of this relationship is equal to *β*. The parameter *β* can therefore be used to characterize the damage evolution rate of the material under a given loading condition.

Based on the above damage evolution equation, a damage model applicable to asphalt materials under cyclic loading can be derived [30,31], as expressed in Equation (15).

(15) $$\ln(\frac{dD}{dN})=\beta \ln(\frac{\partial W_{N}}{\partial N})$$

As indicated by Equation (15), as the fatigue life fraction gradually increases from 0 to 1, damage progressively evolves toward the failure threshold. During this process, the damage predicted by the model is directly governed by the parameter *β* [30,31]. Based on the above analysis, *β* can be regarded as the representative rate of asphalt cracking damage, quantitatively reflecting the rate at which cracking damage develops.

The Arrhenius kinetic equation is expressed in Equation (16), which can accurately describe the evolution law of the rate of representative physical reaction processes at different temperatures [29,32,33].

(16) $$k=Ae^{\frac{-E_{a}}{RT}}$$

where *k* is the rate constant; *A* is the pre-exponential factor; and *R* is the gas constant, in kJ/(mol·K). The authors have employed the Arrhenius equation to establish a relationship between the damage rate and field aging temperature, and developed a kinetic-based damage prediction model to characterize the fatigue process of asphalt materials [34,35]. In the fundamental assumptions of kinetic theory, the rate constant k typically measures the speed at which any physical or chemical process reaches equilibrium. This rate constant k can be obtained experimentally. The minimum energy required to initiate a specific physical or chemical process within a system is termed activation energy. Different physical or chemical processes possess distinct activation energies, and the activation energy for a given process depends solely on the material’s intrinsic properties.

*T* is the absolute temperature, in *K*; and *E_a_* is the activation energy, in J/mol.

For the damage model given in Equation (14), the parameter *β* can be selected as the representative rate of asphalt cracking damage. By combining Equations (15) and (16), a kinetics model for asphalt cracking damage can be obtained, as follows:

(17) $$D=1-{\left(1-\frac{N}{N_{f}}\right)}^{\frac{1}{1+2Ae^{\frac{-E_{a}}{RT}}}}$$

where *E*_a_ represents activation energy for cracking damage.

The proposed model was employed to fit the damage curves of the alkali-treated WSVF-modified asphalt, the LF-modified asphalt, and base asphalt, and the fitting results are presented in Figure 5 and Table 2. As shown in Figure 5 and Table 2, the damage model expressed by Equation (14) can accurately capture the cracking damage evolution of the alkali-treated WSVF-modified asphalt, the LF-modified asphalt, and 70# gel-like base asphalt under cyclic loading. As shown in Equation (16), the well-established model can describe the overall trend of asphalt damage from a phenomenological perspective, but it struggles to quantify the rate of damage progression and intrinsic resistance at the energy level. This study proposes a fatigue damage dynamics model based on a mechanical energy balance framework. By introducing the dynamic parameters *k* (damage rate constant) and *E_a_* (damage activation energy), it not only characterizes the damage state, but also quantitatively describes the speed of damage progression and the energy barrier for damage initiation. This establishes a theoretical link between macroscopic damage behavior and microscopic energy dissipation mechanisms beyond classical models. This kinetic framework provides a novel quantitative analytical pathway for deepening the understanding of asphalt fatigue damage mechanisms, demonstrating clear theoretical necessity and scientific value.

As indicated in Table 2, as the temperature decreases sequentially from 25 °C to 20 °C, and then to 15 °C, the representative cracking damage rate parameters of the alkali-treated WSVF-modified asphalt, the LF-modified asphalt, and 70# gel-like base asphalt exhibit decreasing trends, while the corresponding fatigue life *N_f_* values show increasing trends. These results demonstrate that a reduction in temperature can effectively retard the propagation rate of fatigue cracks in asphalt.

### 2.4. Activation Energy Analysis of Fatigue Cracking in Alkali-Treated WSVF-Modified Asphalt

According to kinetics theory [29,32,33], the cracking damage activation energy represents the minimum energy required for cracking damage to occur in asphalt. A higher cracking damage activation energy indicates a higher energy threshold for the initiation of cracking damage, meaning that the cracking damage process is less likely to occur. Therefore, by comparatively analyzing the differences in cracking damage activation energy between the alkali-treated WSVF-modified asphalt and base asphalt, the toughening effect of WSVF on asphalt can be characterized.

**Figure 6:**
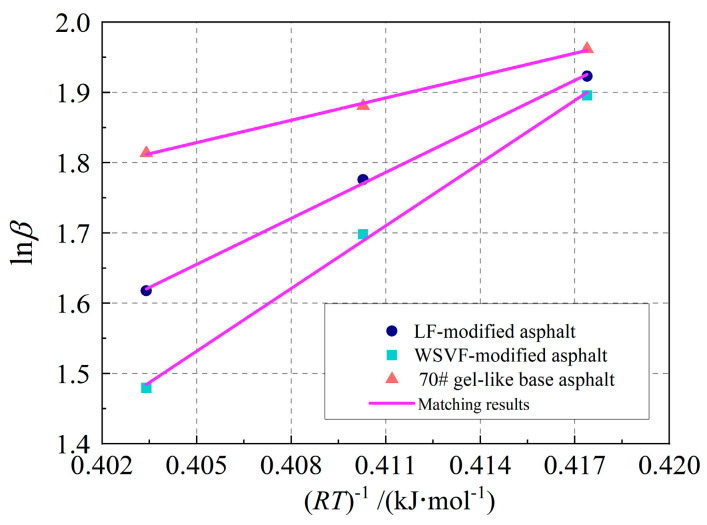
Matching results of Arrhenius kinetics equation.

**Figure 7:**
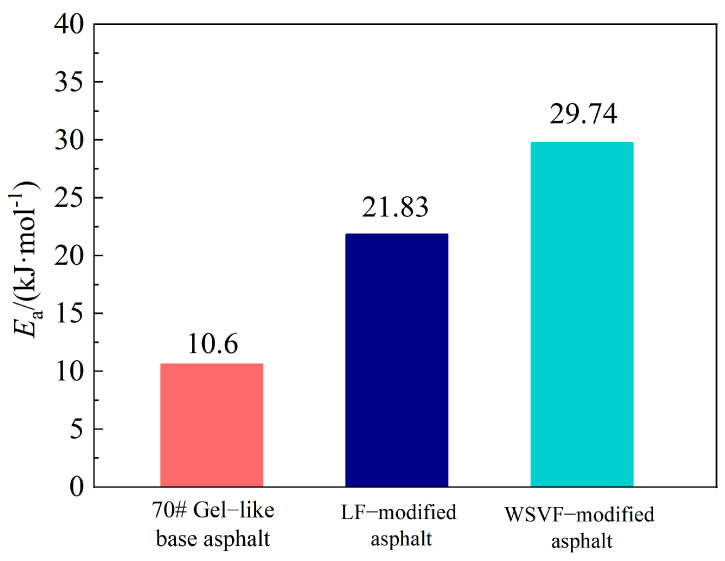
Activation energy for cracking damage.

To determine the cracking damage activation energy of the alkali-treated WSVF-modified asphalt and base asphalt, the representative cracking damage rates at different temperatures were substituted into the Arrhenius kinetics equation in double-logarithmic coordinates, as expressed in Equation (18). Equation (18) was used to fit the representative cracking damage rates under different temperature conditions, as shown in Figure 6. As illustrated in Figure 6, the Arrhenius kinetics equation can accurately fit the representative cracking damage rates under various conditions, further demonstrating the effectiveness of using the Arrhenius model to quantify the cracking damage activation energy of the alkali-treated WSVF-modified asphalt and base asphalt. The absolute value of the slope of the fitted straight line in Figure 6 was taken as the cracking damage activation energy of the alkali-treated WSVF-modified asphalt and base asphalt, and the results are shown in Figure 7.

(18) $$\ln\left(Ae^{\frac{-E_{a}}{RT}}\right)=\ln A-\frac{E_{a}}{RT}$$

As shown in Figure 7 and Table 3, the cracking damage activation energy of the 70# gel-like base asphalt is 10.60 kJ·mol^−1^, which is lower than that of the LF-modified asphalt (21.83 kJ·mol^−1^) and the alkali-treated WSVF-modified asphalt (29.74 kJ·mol^−1^). The minimum energy values required to induce cracking damage in the alkali-treated WSVF-modified asphalt and the LF-modified asphalt were 19.14 kJ·mol^−1^ higher than that of the base asphalt, indicating that incorporating an appropriate amount of WSVF into 70# gel-like base asphalt increases the difficulty of cracking damage initiation and prolongs its service life. This improvement is mainly attributed to the grooved surface structure of the alkali-treated WSVF, which provides excellent asphalt adsorption and storage capacity, resulting in strong interfacial bonding between the fiber and asphalt and, thereby, achieving an effective toughening effect. Furthermore, the minimum energy required for cracking damage in the alkali-treated WSVF-modified asphalt is 7.91 kJ·mol^−1^ higher than that of the LF-modified asphalt, indicating that WSVF exhibits a superior toughening effect on 70# gel-like base asphalt compared with LF. This advantage is primarily due to the relatively smooth surface of lignin fiber without chemical treatment, whose surface roughness and oil absorption capacity are significantly lower than those of the NaOH-treated WSVF.

## 3. Conclusions

(1) Based on the energy–mechanical equilibrium equations of true asphalt and apparent asphalt, a cracking volume model for asphalt can be established. For the alkali-treated WSVF-modified asphalt, the cracking volume response under cyclic loading exhibits three nonlinear stages.

(2) The cracking volume can be used to scientifically define the damage variable of the alkali-treated WSVF-modified asphalt. The parameter *β* in the damage evolution equation can serve as the representative rate of asphalt cracking damage, enabling quantitative evaluation of the overall development rate of cracking damage. Compared with 70# gel-like base asphalt, the *k* values of WSVF-modified asphalt at 15 °C, 20 °C, and 25 °C decreased by 38.3%, 16.7%, and −8.5%, respectively, demonstrating that WSVF exhibits a superior ability to slow down the cracking damage process, particularly at intermediate and low temperatures.

(3) The established fatigue damage kinetics model can accurately describe the fatigue damage evolution process of the alkali-treated WSVF-modified asphalt. The fatigue life values of WSVF-modified asphalt at 15 °C, 20 °C, and 25 °C increased by 33.4%, 34.3%, and 33.4%, respectively, compared to 70# gel-like base asphalt. These results indicate that WSVF significantly enhances the fatigue resistance of asphalt, with an average improvement of approximately 33.7% over base asphalt.

(4) A higher cracking damage activation energy indicates that a greater amount of energy is required for cracking damage to occur. The cracking damage activation energy values of the alkali-treated WSVF-modified asphalt and 70# gel-like base asphalt are 29.14 kJ·mol^−1^ and 10.60 kJ·mol^−1^, respectively. After alkali treatment, the surface of WSVF exhibits grooves, providing excellent asphalt adsorption and storage capacity. Moreover, the fibers interconnect through the adhesive action of asphalt to form a three-dimensional network skeleton structure, thereby increasing the cracking damage activation energy of asphalt and achieving an effective toughening effect.

This study proposes a method for evaluating the toughening effect of discarded sweet potato vine fiber on base asphalt. However, the theoretical framework exhibits certain limitations. Primarily, the macroscopic apparent *E_a_* employed essentially equates the multiphase, multi-mechanism damage process of asphalt to an “effective average value,” thereby simplifying the actual distributed damage evolution system. Furthermore, this “damage activation energy” is not a material constant. Its value is influenced by both loading conditions and damage stages, making it a condition-dependent parameter within a specific experimental framework, rather than one solely determined by intrinsic material properties. While these simplifications aid in establishing preliminary kinetic correlations, they also highlight the model’s limitations in mechanism resolution and universality. Based on these insights, subsequent research will proceed along the following directions: First, we will aim to validate the applicability of this method to other plant fibers and conduct systematic sensitivity analysis of fiber content to clarify the nonlinear characteristics of toughening effects, as well as the optimal content range. Second, we will introduce quantitative interface characterization techniques, such as atomic force microscopy roughness mapping and surface energy analysis, to establish predictive models linking microscopic interface parameters to macroscopic fatigue performance. Finally, by integrating multidimensional experimental data with theoretical insights, we will develop an intelligent decision-making system for engineering applications, establishing an environmentally sustainable, high-performance, and economically viable modified asphalt technology system.

## 4. Materials and Methods

### 4.1. Materials and Specimen Fabrication

#### 4.1.1. Gel-like Base Asphalt

In this study, 70# gel-like base asphalt was selected as the binder for preparing the waste sweet potato vine straw fiber (WSVF)-modified asphalt. According to classical colloid theory, the microstructure of asphalt can be classified into sol, gel, and gel-like types, based on the degree of aromatization and the dispersion state of asphaltenes. The 70# base asphalt used in this study exhibits a typical gel-like structure at service temperatures. This structure is characterized by a continuous three-dimensional network of asphaltenes, which endows the asphalt with enhanced elasticity and resistance to permanent deformation. At elevated temperatures (above 100 °C), this network transitions to a more sol-dominant state, while, below 50 °C, the gel-like characteristics become pronounced again. The basic technical indices of the 70# gel-like base asphalt are listed in Table 4. By comparison with the technical requirements specified in the relevant standard [36], the asphalt grade is confirmed to meet the specification requirements.

#### 4.1.2. Preparation of Alkali-Treated Sweet Potato Vine and Straw Fibers

**Figure 8:**
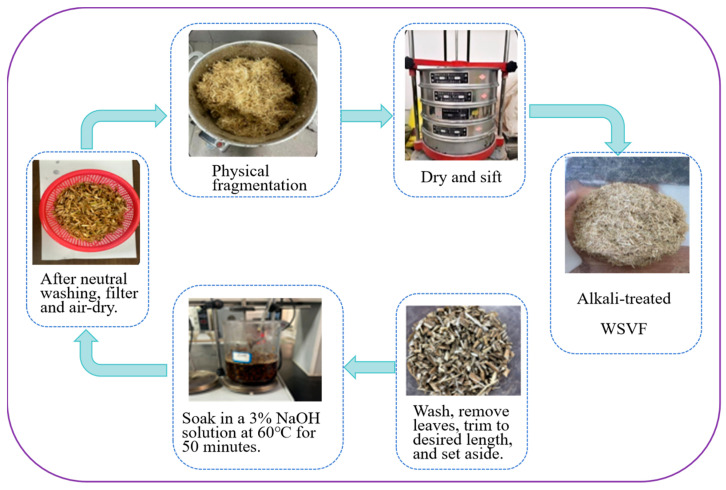
Recycling sweet potato vines for preparing alkali-treated modified asphalt using WSVF.

According to the preparation procedure for WSVF proposed in Ref. [17] (as shown in Figure 8), WSVF in its natural state was first pretreated, including leaf removal and washing to eliminate surface dust and other water-soluble impurities. After wiping dry, the straw was cut into small segments, approximately 10 mm in length, and then oven-dried. Subsequently, a NaOH solution with a mass fraction of 3% was prepared, and the straw segments were immersed in the NaOH solution at 60 °C for 50 min. After treatment, the fibers were washed to neutrality, followed by crushing and drying, and finally sieved to obtain the required WSVF. Under the preparation process of 3% NaOH concentration and 60 °C treatment for 50 min, the fiber extraction rate and oil absorption capacity achieved optimal equilibrium. The fiber diameter decreased, the aspect ratio increased, and the specific surface area significantly improved. The treated fiber surface exhibited numerous fine, dense grooves and undulating textures. This not only substantially enhances the fiber’s physical adsorption and storage capacity for asphalt, but also creates a mechanical interlocking effect with the asphalt matrix. This provided excellent interfacial bonding conditions for subsequent asphalt modification.

#### 4.1.3. WSVF-Modified Asphalt

**Figure 9:**
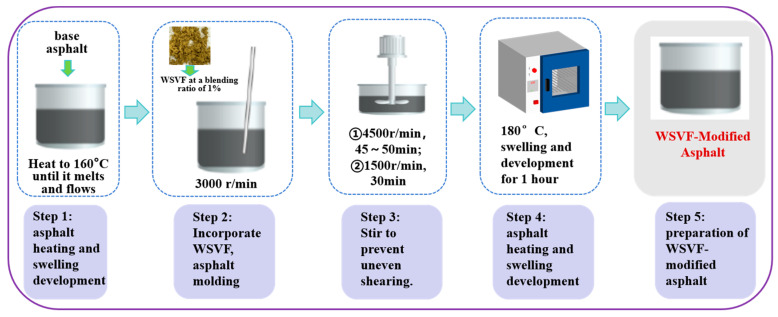
Flowchart for preparing fiber-modified asphalt using WSVF.

According to the preparation process of the WSVF-modified asphalt determined in Ref. [19] (as shown in Figure 9), rheological performance tests indicated that the optimal content of WSVF is 1% by mass of the asphalt. The selected LF is a commercially available product, without any additional surface treatment. Its specifications and performance comply with current industry standards for road engineering applications. Based on practical construction experience with road fibers and comprehensive evaluation, it was determined that a lignin fiber content of approximately 1% yields favorable overall performance, including balanced high- and low-temperature properties. Therefore, the lignin fiber content in the modified asphalt was set at 1% [19].

First, the 70# gel-like base asphalt was placed in an electric blast drying oven and heated to 160 °C until it reached a molten and flowable state. Subsequently, at a rotational speed of 3000 r/min, pre-weighed WSVF amounting to 1% of the asphalt mass was slowly and uniformly added. Then, the rotational speed was increased to 4500 r/min and maintained for 45–50 min of continuous shearing. During this process, constant manual stirring with a glass rod was required to ensure uniform shearing. After that, the rotational speed was reduced to 1500 r/min, and shearing was continued for 30 min, allowing the fibers to disperse more fully under low-speed mixing and to be incorporated into the asphalt in a finer state. Next, the asphalt was swollen and developed in an oven at 180 °C for one hour, at which point it adopted a gel-like structure, predominantly in the sol state. Finally, the asphalt was cooled to approximately 15 °C to produce the WSVF-modified asphalt. At this point, the asphalt exhibited a gel-like structure, where the gel state was more pronounced than the sol state.

### 4.2. Test Methods

In this study, 70# gel-like base asphalt and LF-modified asphalt were used as reference materials to evaluate the fatigue properties of WSVF-modified asphalt under stress-controlled mode. The fatigue tests were conducted at a loading frequency of 10 Hz, with five parallel specimens prepared for each condition. Previous studies [37] have shown that asphalt fatigue cracking mainly occurs in the intermediate temperature range of 10 °C to 30 °C; therefore, most related fatigue tests were performed within this temperature range. Accordingly, 25 °C, 20 °C, and 15 °C were selected as the test temperatures for evaluating the fatigue performance of asphalt in this study. At these temperatures, the tested asphalt exhibited a gel-like structure, where the gel state was more pronounced than the sol state.

#### 4.2.1. Linear Amplitude Time Sweep (LATS) Test

The LATS test was employed to quantify the critical nonlinear viscoelastic shear stress (*τ*_cri_). A load-controlled mode was applied using a strain-controlled standard sinusoidal load, in which the shear strain amplitude increased linearly from 0.001 to 0.3. Taking the LATS tests of the WSVF-modified asphalt and the LF-modified asphalt at 20 °C as examples, the determination method of *τ*_cri_ was analyzed.

**Figure 10:**
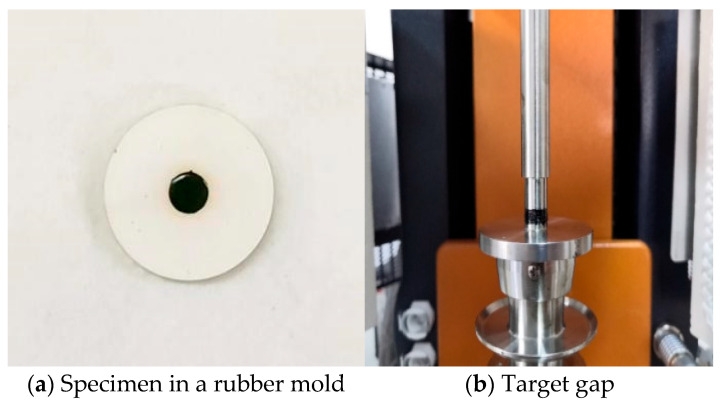
Preparation of standard DSR specimens for fatigue testing.

A dynamic shear rheometer (DSR) (Anton Paar, Graz, Austria) was used as both the specimen preparation and testing platform for the 70# gel-like base asphalt, LF-modified asphalt, and WSVF-modified asphalt. The preparation procedure was as follows. First, the molten asphalt was poured into a cylindrical rubber mold to preliminarily form a columnar asphalt specimen. Subsequently, the specimen was placed in the DSR for final shaping, with the trimming gap and the target gap set to 2050 μm and 2000 μm, respectively. The resulting asphalt specimen was a cylinder with a diameter of 8 mm and a height of 2 mm (as shown in Figure 10). This procedure was applicable to the preparation of specimens for both the 70# gel-like base asphalt and the WSVF-modified asphalt. Fabrication of cylindrical asphalt specimens (8 mm diameter × 2 mm height) using a rubber mold and DSR ensured dimensional consistency for subsequent time sweep tests (LATS, NDTS, and DTS).

**Figure 11:**
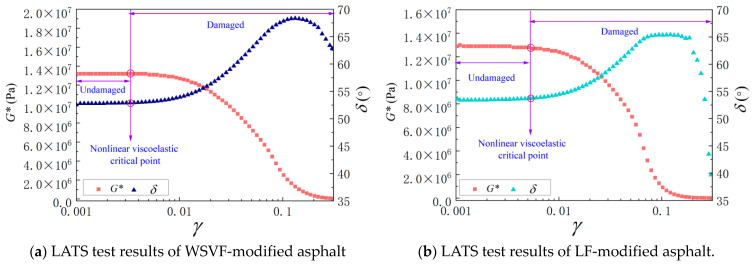
LATS test results of asphalt.

As shown in Figure 11, when the loading amplitude is below the critical point corresponding to the nonlinear viscoelastic critical shear strain (*γ*_cri_) and critical shear stress (*τ*_cri_), no damage accumulation occurs in the material, and both *δ* and G* remain in a quasi-linear steady state [38,39,40]. As the loading level increases, progressive damage accumulation causes *δ* to increase initially and then decrease, while G* exhibits a nonlinear downward trend. The shear strain corresponding to the intersection point at which G* drops sharply and the linear steady state terminates is defined as *γ*_cri_. The shear stress corresponding to *γ*_cri_ is recorded as *τ*_cri._ Accordingly, the *τ*_cri_ values of the WSVF-modified asphalt and the LF-modified asphalt at 20 °C were 87 kPa and 83 kPa, respectively.

#### 4.2.2. Non-Damage Time Sweep (NDTS) Test

**Figure 12:**
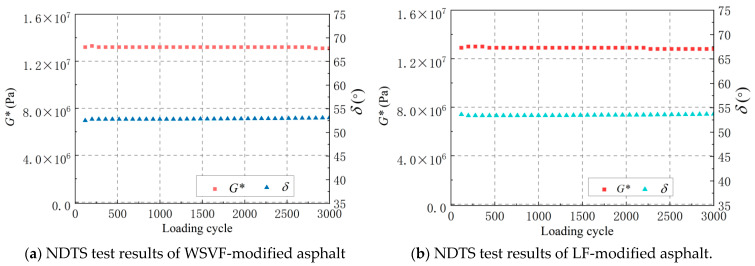
NDTS test results of asphalt.

The non-damaging mechanical responses of the tested asphalts were analyzed using a stress-controlled NDTS test. A standard sinusoidal load was applied, with the stress amplitude set to *τ*_cri_ and a loading duration of 300 s. Figure 12 presents the test results of the phase angle (*δ*) and complex shear modulus (G*) for the WSVF-modified asphalt and the LF-modified asphalt at 20 °C. Under cyclic loading at the *τ*_cri_ amplitude, neither *δ* nor G* exhibited significant changes for either modified asphalt, indicating that the asphalts remained in undamaged states throughout the loading process at *τ*_cri_ [41,42].

#### 4.2.3. Damage Time Sweep (DTS) Test

**Figure 13:**
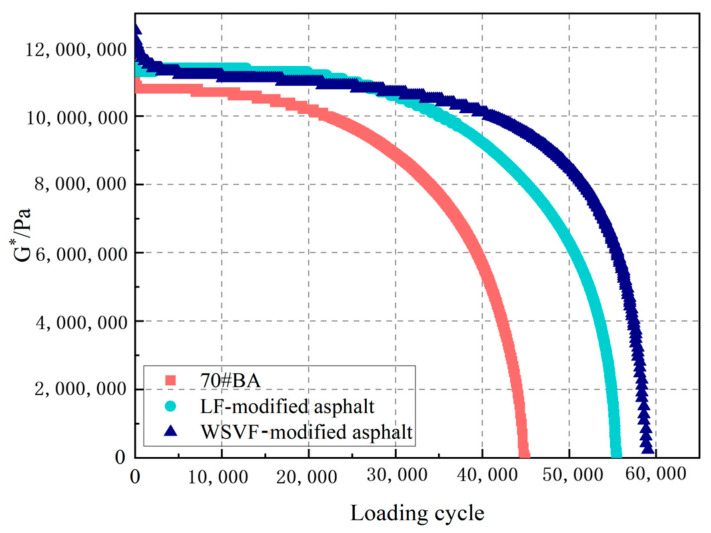
DTS Test results of the WSVF-modified asphalt, LF-modified asphalt, and 70# base asphalt.

The damage-related mechanical responses of 70# base asphalt, lignin fiber-modified asphalt, and WSVF-modified asphalt were analyzed using dynamic thermomechanical testing (DTS). First, the asphalt specimens subjected to the NDTS test were allowed to recover for 300 s. Subsequently, the same specimens were loaded under a stress-controlled standard sinusoidal load, with the stress amplitudes listed in Table 5. Taking the tests conducted at 20 °C and 100 kPa for the 70# base asphalt, the LF-modified asphalt, and the WSVF-modified asphalt as examples, the decay trend of G* was described. Figure 13 shows the corresponding G* test results. The complex shear modulus exhibited a progressive nonlinear decay, which is attributed to the applied stress amplitude exceeding the critical shear stress *τ*_cri_, leading to cracking damage accumulation during cyclic loading. In addition, the G* decay rate of the WSVF-modified asphalt was lower than those of the 70# gel-like base asphalt and the LF-modified asphalt, indicating that WSVF can effectively retard the damage evolution rate of asphalt. Asphalt is a typical temperature-sensitive viscoelastic material whose mechanical behavior strongly depends on temperature and loading duration. High modulus with low phase angle indicates an elastic/glassy state (low temperatures), while low modulus with high phase angle signifies a viscous/flowing state (high temperatures). The intermediate temperature range represents a typical viscoelastic transition zone. Within the 15–25 °C intermediate temperature range, asphalt achieves optimal viscoelastic equilibrium. Its modulus is moderate, simultaneously exhibiting elastic (energy storage) and viscous (energy dissipation) components. The dominant failure mechanisms—low-temperature brittle fracture and high-temperature flow—are mitigated in this temperature window. Consequently, the relatively mild yet cyclic loading-dependent fatigue damage mechanism becomes predominant, allowing cracks to nucleate stably and propagate progressively.

#### 4.2.4. Scanning Electron Microscopy (SEM) Test

**Figure 14:**
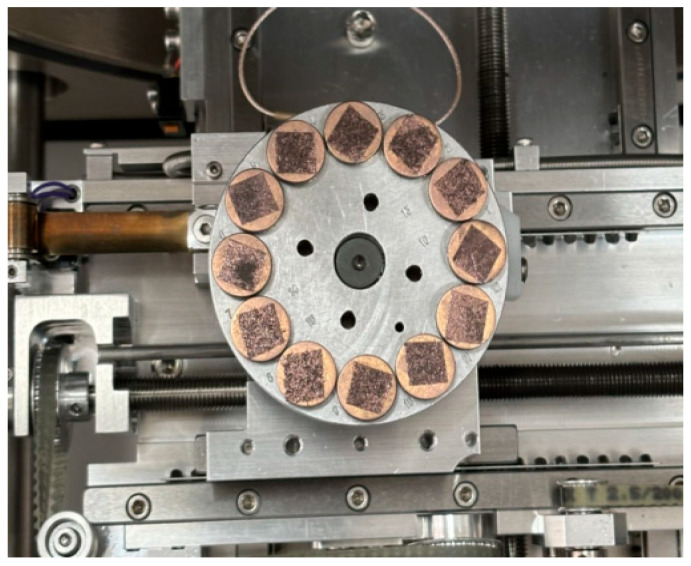
SEM sample preparation.

The micro-morphology of the WSVF-modified asphalt was examined using a JSM-7500F field-emission SEM (JEOL Ltd., Tokyo, Japan). The preparation procedure of the test specimens is illustrated in Figure 14. Owing to the insulating nature of the WSVF-modified asphalt, gold sputtering was required prior to SEM observation. After the sample surface was fully coated with a conductive metal layer, microscopic observation was conducted. The specimen dimensions were approximately 10 mm × 10 mm × 10 mm.

## Figures and Tables

**Table 1:** Loading amplitudes and fatigue life of destructive time sweep test.

Asphalt Type	Temperature/°C	Loading Amplitude/kPa	Fatigue Life	Coefficient of Variation/%
70# gel-like base asphalt	15	250	79,410	8.537
	20	100	45,040	8.701
	25	80	25,800	9.057
Lignin-modified asphalt	15	250	97,880	11.633
	20	100	55,466	10.816
	25	80	32,022	10.698
WSVF-modified asphalt	15	250	105,880	10.253
	20	100	60,466	11.031
	25	80	34,422	10.845

**Table 2:** Fitting results of damage model.

Type of Asphalt	Temperature/°C	$k=Ae^{\frac{-E_{a}}{RT}}$	*R* ^2^
70# gel-like base asphalt	15	7.11267	0.891
	20	6.55815	0.907
	25	6.13306	0.936
LF-modified asphalt	15	5.04135	0.914
	20	5.90578	0.903
	25	6.84256	0.892
WSVF-modified asphalt	15	4.39037	0.998
	20	5.46433	0.874
	25	6.65687	0.912

**Table 3:** Results of fatigue damage activation energy.

Type of Asphalt	Activation Energy kJ/mol	Coefficient of Variation/%
70# gel-like base asphalt	10.60	11.178
LF-modified asphalt	21.83	13.047
WSVF-modified asphalt	29.74	11.962

**Table 4:** Basic performance indices of asphalt.

Test Item	Unit	Test Result	Technical Requirement
Penetration (25 °C, 100 g, 5 s)	0.1 mm	67.3	60~80
Ductility (5 cm/min, 10 °C)	cm	27.7	≮15
Softening point (Ring-and-Ball method)	°C	45.6	≮46

**Table 5:** Load amplitudes and fatigue life of destructive time sweep test.

Type of Asphalt	Temperature/°C	Load Amplitude (kPa)
70# gel-like base asphalt	15	250
	20	100
	25	80
LF-modified asphalt	15	250
	20	100
	25	80
WSVF-modified asphalt	15	250
	20	100
	25	80

## Data Availability

Data are contained within the article. Some or all of the data, models, or code that support the findings of this study are available from the corresponding authors upon reasonable request.
